# Attention to electronic prescription process improves time to first-dose antibiotics in patients on the ICU

**DOI:** 10.1186/cc9637

**Published:** 2011-03-11

**Authors:** R Wan, D Gonzalez Bermejo, S Moore, C Whiteley, C Mckenzie, A Jones

**Affiliations:** 1 Guy's & St Thomas' NHS Foundation Trust, London, UK; 2 La Paz University Hospital, Madrid, Spain; 3 Kings College Medical School, London, UK

## Introduction

Effective timely antibiotic administration is associated with increased survival to discharge in patients with septic shock [1]. Time to antibiotic administration was the strongest predictor of outcome and is a key recommendation in sepsis management [2]. However, implementation faces barriers at clinician, patient and environmental levels [3].

## Methods

A retrospective review of antibiotic prescribing on a 30-bed university medical-surgical ICU. Data were extracted from the clinical informatics system (Intellivue Clinical Portfolio (ICIP) Philips). For a 4-month period (baseline assessment September 2009 to January 2010), patients initiated on new intravenous antibiotics were included. After baseline data review, the ICIP prescription order process was modified to automatically include STAT doses. A further 4-month period (post implementation) review followed.

## Results

At baseline, 139 patients and 320 prescriptions were analysed. Median time to antibiotic administration was 127 minutes (IQR 29 to 272). The proportion of antibiotics administered within 1 hour and 3 hours was found to be 81/320 (25%) and 193/320 (60%), respectively. Analysis by antibiotic class revealed aminoglycosides and vancomycin had the lowest median time that in our unit are initiated as STAT doses, 86 minutes (IQR 43 to 195 minutes). Post modification of the ICIP prescription order process, 139 patients and 194 prescriptions were analysed. Median time to antibiotic administration improved to 79 minutes (IQR 43 to 159), *P *< 0.0001. A greater proportion was administered within 1 hour (70/194, 37%) and 3 hours (153/194, 79%), *P *≤ 0.001, for this cohort.

## Conclusions

Barriers to timely administration of antibiotics exist, an intervention shown to significantly improve patient outcome. This study demonstrates modification of an electronic prescribing order process contributes to improved performance. However, a multifactorial problem may exist. It confirms clinical informatics systems play in improving the delivery of quality patient care in the ICU.
